# High expression of cytokeratin CAM5.2 in esophageal squamous cell carcinoma is associated with poor prognosis

**DOI:** 10.1097/MD.0000000000017104

**Published:** 2019-09-13

**Authors:** Shujin He, Jie Peng, Lei Li, Ying Xu, Xiaoxiao Wu, Juan Yu, Jianli Liu, Jinguo Zhang, Renya Zhang, Wei Wang

**Affiliations:** a Department of Pathology; b Department of Cardiovasology, Affiliated Hospital of Jining Medical University, Jining Medical University, Jining, Shandong, China.

**Keywords:** CAM5.2, esophageal squamous cell carcinoma, prognosis

## Abstract

Esophageal cancer is a common human malignant tumor with high mortality. Glandular epithelial markers, such as CAM5.2, can be expressed in esophageal squamous cell carcinoma (ESCC), but the clinical significance of these cells in ESCC remains elusive.

Immunohistochemical analysis of CAM5.2 was performed on 604 ESCC specimens using tissue microarray. Our study design and study population used retrospective cohorts based on the hospital information system and pathological information management system which included medical information, date of admission, procedures undergone, registration, examinations, and medication.

In total, positive staining of CAM5.2 was 145 of 604 (24%). Statistical analysis showed that the expression of CAM5.2 had no relationship with sex, age, tumor differentiation, tumor size, tumor-node-metastasis (TNM) classification, and lymph node metastasis, but it was significantly associated with poor prognosis of overall survival (*P* = .0041) and disease-free survival (*P* = .0048) in ESCC patients.

Herein, we report for the first time that the high expression of the CAM 5.2 is an independent predictor of poor prognosis in patients with ESCC.

## 1 Introduction

Esophageal cancer is the 7th most common cancer among males and among both sexes combined in the world and ranks 6th in terms of mortality overall because of the poor survival rate it confers.^[1,2]^ Compared with more developed geographic regions, overall incidence rates are 2-fold higher in less-developed countries, with the highest rates occurring in Asia.^[1]^ Moreover, incidence and mortality rates in males are 2- to 3-fold higher than the rates in females.^[1]^ Esophageal squamous cell carcinoma (ESCC) is the predominant histologic type with the highest incidence rate in populations within Southeastern and Central Asia.^[2]^ With various treatment methods employed in clinical practice after extensive research, the diagnosis and treatment of ESCC have been greatly improved. However, the prognosis remains poor, with 5-year survival proportions of 21% and 14% (2005–2011) in the United States for whites and blacks, respectively, and 12% (2000–2007) in Europe,^[2]^ which is far below the estimated effectiveness of the therapy. Accumulating evidence suggests that the prognosis is affected by several factors, including the delayed diagnosis, high recurrence, and metastasis rate. To more precisely delineate the major molecular mechanism of initiation and progression, much effort has been invested recently in various types of cancer stem cells.^[3]^ Thus, identifying the diagnostic and prognostic tumor markers and further elucidating their clinical implications are urgently needed. Therefore, the study of tumor markers is of great significance in the diagnosis and treatment of cancer.^[4–7]^

Cytokeratin cocktail, an anti-CAM5.2 reagent, identifies CK7 and CK8,^[8,9]^ which are typically used to identify secretory epithelial (glandular epithelium) cells. The CAM5.2 antibody reacts with K8 and K18 was highlighted by Makin et al. in 2009,^[10]^ so CAM5.2 has been mistakenly equated to K8/18 monoclonal antibody. The authors may have inadvertently annotated that CAM5.2 antibody reacts with CK8 and CK18.^[11]^ For BD Biosciences, CAM5.2 (clone CAM5.2) reagent reacted with human cytokeratin intermediate filament proteins 48 and 52 KD, and was identified as CK7 and CK8, respectively. Authors pointed out that anticytokeratin CAM5.2 is specific for CK8 and to a lesser extent, for the closely related CK7, but shows no reactivity with CK18 or CK19.^[8,11–13]^

Although rarely expressed in squamous cell carcinoma, published work has reported that CK7 can be expressed in squamous epithelium of ESCC patients, and the relationship between CK7 and prognosis of different clinical stages in patients was observed.^[14,15]^ The relationship of CK8 expression with ESCC has never been reported. CK8 is a luminal marker, and mice lacking the prostate epithelial androgen receptor have increased apoptosis in epithelial CK8-positive luminal cells and increased proliferation in epithelial CK5-positive basal cells.^[16]^

In this study, CAM5.2 expression was detected in 604 ESCC samples. We characterized CAM5.2 expression in ESCC tissues and evaluated the association between CAM5.2 expression and clinicopathological parameters as well as prognosis.

## 2 Materials and methods

### 2.1 Dataset

The global gene expression profile data set GDS3838 (https://www.ncbi.nlm.nih.gov/sites/GDSbrowser?acc=GDS3838) was collected from the Gene Expression Omnibus (GEO). The dataset contains 17 ESCC and 17 adjacent normal tissue samples from patients in China,^[17]^ examined with the Affymetrix Human Genome U133A 2.0 Array. All the samples were histologically confirmed and processed by laser-captured microdissection to enrich the epithelial cells before total RNA extraction.

### 2.2 Esophageal biopsy specimens

**Figure 1:**
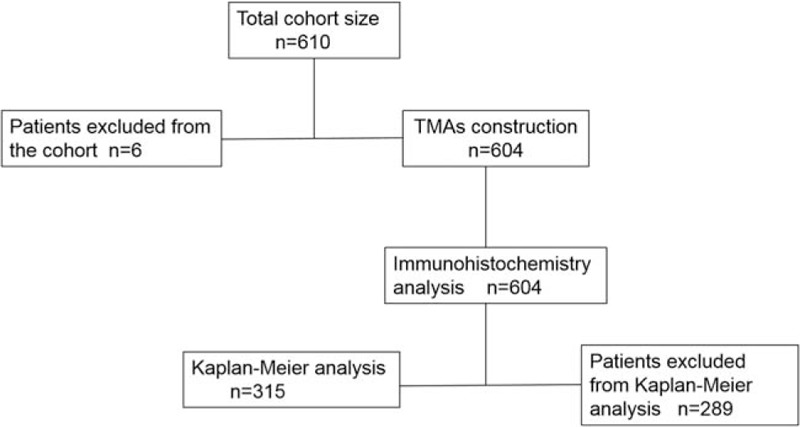
The flow of case cohort. A total of 604 cases were identified, and only 315 of them were included in Kaplan-Meier analysis.

A cohort of 604 subjects with ESCC were recruited after excluding 6 recipients according to the exclusion criteria between 2008 and 2014 from the Affiliated Hospital of Jining Medical University (Shandong, PR China). Data were censored on December 31, 2016. Patients who were diagnosed with ESCC by June 31, 2014 were eligible for tissue microarray (TMA) construction and further immunohistochemical staining, whereas only patients with a follow-up record were included in the study for overall survival (OS, Fig. 1). The inclusion criteria were as follows: ESCC was clinically diagnosed and confirmed by pathological diagnosis after operation; patients without other cancer history or family history. Exclusion constituted: postoperative pathological findings were nonesophageal cancer; patients with other cancer history and family history; patients who did not agree to participate in this study. All specimens were immediately fixed in 4% buffered paraformaldehyde, routinely processed, and embedded with paraffin. Tumors were classified according to standard TNM staging guidelines of UICC (TNM Classification of Malignant Tumors Eighth edition). The study protocol was reviewed and approved by the local ethics committee. The study was approved by the ethics committee of Jining Medical University.

### 2.3 TMA construction

Representative areas of the ESCC were marked on each hematoxylin-eosin slide and tissue paraffin block, and the marked areas of tissue paraffin blocks were sampled for the TMAs. The TMAs were assembled with a tissue-arraying instrument (Beecher Instruments, Silver Springs, MD). As described by Kallioniemi et al,^[18]^ 1 typical core was taken from each patient sample, and the diameter of each core was 2 mm in TMAs.

### 2.4 Immunohistochemical staining

Immunohistochemical staining of the cytokeratin (CAM5.2) protein was performed on the TMA slides using the streptavidin-peroxidase (S-P) method as previously described with minor modifications.^[19]^ Briefly, each TMA section was deparaffinized and rehydrated, and antigen retrieval was done at 95°C in 1× ethylenediaminetetraacetic acid buffer (pH 9.0) for 15 minutes. Inactivation of endogenous peroxidase was performed using 0.3% H_2_O_2_-methanol for 30 minutes. Nonspecific binding was prevented by incubation with normal serum for 20 minutes at room temperature (RT), followed by incubation with the primary anticytokeratin (CAM 5.2) reagent (BD Biosciences, Cat. No. 349205) at 4°C overnight. Antibody binding was detected using Envision reagents (Dako REAL EnVision Detection System; peroxidase/DAB1, Dako Cytomation, Denmark). The immune reaction was visualized by incubation with 3, 30-diaminobenzidine-chro-mogen-subtrate (DAB1 Chromogen, DAKOVR, Carpinteria, CA) for 10 minutes at RT. Finally, slides were counterstained with hematoxylin, dehydrated, and coverslipped with an automatic sealing machine (Sakura GLC 550, Tissue-TekVR, Alphen aanden Rijn, The Netherlands). CAM5.2 immunoreactivity scores were measured by 2 independent pathologists according to both the intensity and extent of staining without previous knowledge of patients’ clinicopathological characteristics. Briefly, in the tumor, the assessment was classified as follows: immunopositive cells were counted respectively in 5 randomly selected microscopic visual fields per well. The number of positive cells <5% was judged as 0, 5 to 25% as 1, 26% to 50% as 2, 51% to 75% as 3, and 76% to 100% as 4. Regarding the score of positive staining intensity, colorlessness was judged to be 0, light yellow to be 1, brownish yellow to be 2, and tan to be 3. The sum of the 2 scores is the positive grade; 0 to 3 score was defined as low expression and 4 to 7 score as high expression.^[20,21]^

### 2.5 Follow-up

Patients underwent long-term follow-up, and the mean survival time was 29 months and the median follow-up time was 24 months (1–95.2 months) by telephone until death or the final follow-up. Among the 604 patients, 289 patients were lost to follow-up, and only 315 participated (182 patients died at the final follow-up, and 26 patients progressed during follow-up).

### 2.6 Statistical analysis

Differences in quantitative variables between groups were analyzed by the Student *t* test. Combined strategies were used to analyze the datasets (201820_at/CK5, 209125_at/CK6a, 213680_at/CK6b, 214580_x_at/CK6c, 209016_s_at/CK7, 209016_s_at/ CK7, 209008_x_at/ CK8) gained from the ESCC GEO profiles GDS3838. The expression matrix was downloaded and processed by statistical methods. Briefly, Log (base 2) expression measures for each probe set were computed using robust multi-array average according to a previous report.^[22]^ The values of *CK5*, *CK6a*, *CK6b*, *CK6c*, *CK7*, and *CK8* genes expression in the 17 ESCC and 17 adjacent normal tissue samples were calculated by single-tail test. The Pearson *χ*2 test was used to analyze the association of CAM5.2 expression with clinicopathological characteristics using the SPSS 13.0 software package (SPSS Inc, Chicago, IL). Survival curves were drawn using the Kaplan–Meier method and compared using the log rank test. Cox's proportional hazards regression model was performed to identify factors which can affect the OS of ESCC patients. Only a *P* value of <.05 was considered statistically significant.

### 2.7 Definition

ESCC was diagnosed based on histopathologic examination of the specimens. Under light microscopy, a variety of histological characteristics can be identified in different degrees of differentiation. Highly differentiated squamous cell carcinomas presented with obvious keratinization, abundant cytoplasm, and few mitotic figures, whereas most poorly differentiated squamous cell carcinomas have no squamous epithelial arrangement. Cellular pleomorphism can easily be observed, and mitoses are common. Diagnosis of each slide was finished by 2 independent pathologists, and to differ adenocarcinoma from poorly differentiated squamous cell carcinoma, p63 or CK5/6 were detected by immunohistochemistry in some of the cases. The time of OS was calculated from the date of surgery to the last follow-up or until death. The time of disease-free survival (DFS) was calculated from the date of surgery to the date of tumor recurrence (confirmed by imaging findings or biopsies).

## 3 Results

### 3.1 Unbiased analysis of differentially expressed epithelial cell-associated genes in ESCC tissues

**Figure 2:**
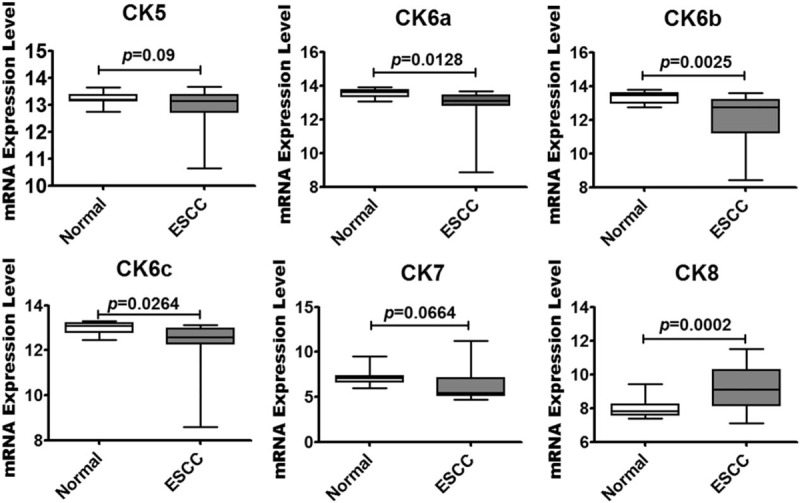
Unbiased analysis of epithelial-associated gene mRNA levels by data mining of the ESCC GEO dataset. Box plot showing the mRNA levels of epithelial-associated molecules in ESCC tissues. These data were collected from the global gene expression profile data set GDS3838, which contains 17 ESCC and 17 adjacent normal tissue samples examined with a Human Genome U133A 2.0 Array from Affymetrix.

First, we analyzed epithelial cell-associated gene expression levels using microarray data collected from the global gene profiling (GEO) dataset GDS3838, which contained the 17 ESCC and 17 adjacent normal tissue samples. The mRNA levels of CK5, CK6a, CK6b, CK6c, CK7, and CK8 were collected from GEO dataset GDS3838. Stratified squamous epithelium makers, such as CK6a, CK6b, and CK6c mRNA levels, were sharply decreased in ESCC samples (Fig. 1), as compared to the levels in their healthy counterparts. However, the mRNA level of glandular epithelium cell marker CK8 was sharply increased in ESCC samples, but the CK7 mRNA level showed no significant difference compared to the levels in their healthy counterparts (Fig. 2), suggesting that the epithelial markers were changed in the tissues of ESCC.

### 3.2 CAM5.2 expression in ESCC patients and its clinicopathological significance

**Figure 3:**
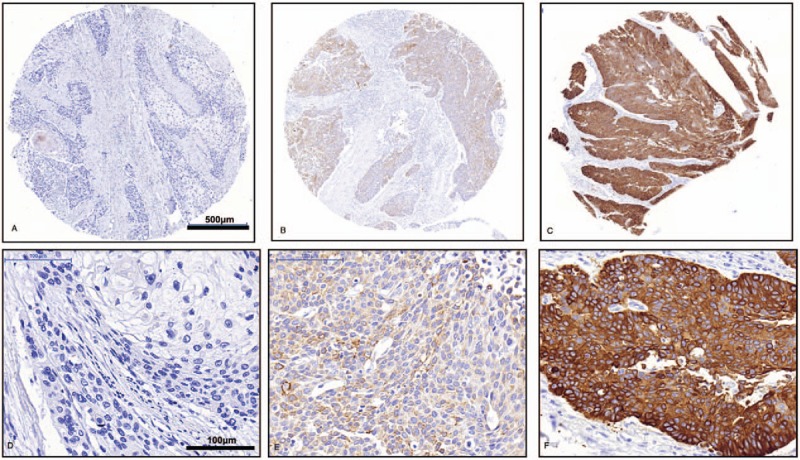
Immunohistochemistry staining for CAM5.2 in ESCC samples. (A and D) CAM5.2-negative staining; (B and E) CAM5.2 weak staining; (C and F) CAM5.2 strong staining. Scale bar: (A, B, C) 500 μm; (D, E, F) 100 μm.

The final number of valid cases was 604, and CAM5.2 strong staining (CAM5.2^H^) was found in 145 cases (145/604, 24%), negative and weak staining (CAM5.2^L^) in 459 cases (459/604, 76%) (Fig. 3 and Table 1). Of the 604 ESCC patients, 470 were male and 140 were female (mean age, 60 years). The difference of CAM5.2 expression in sex, age, tumor differentiation, tumor size, TNM classification, and lymph node metastasis had no statistical significance in the ESCC patients (Table 1).

**Table 1:**
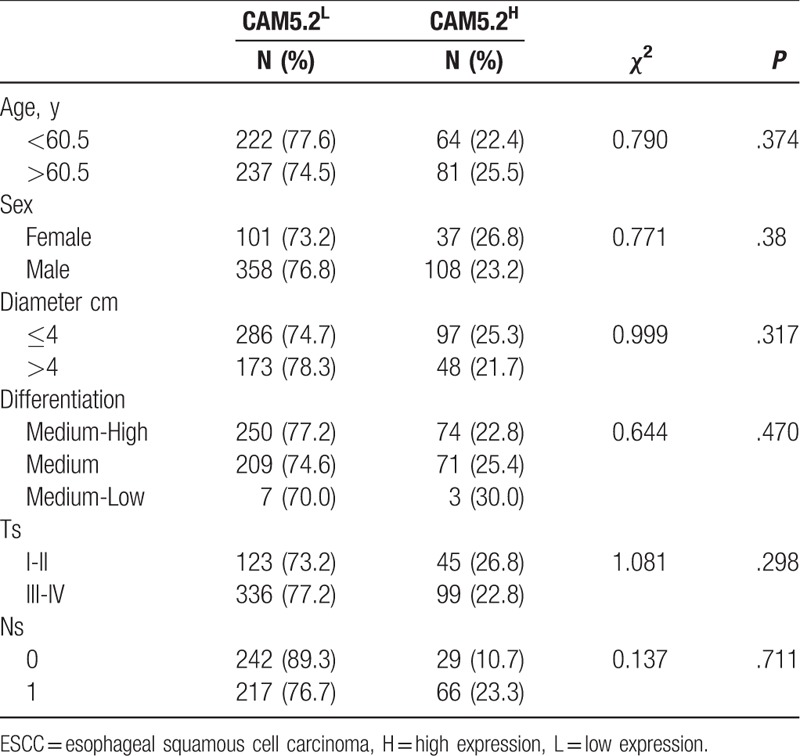
CAM5.2 expression in ESCC patients and its clinicpathological significance, 604 cases.

### 3.3 Strong staining of CAM5.2 predicted poor prognosis of ESCC patients

**Figure 4:**
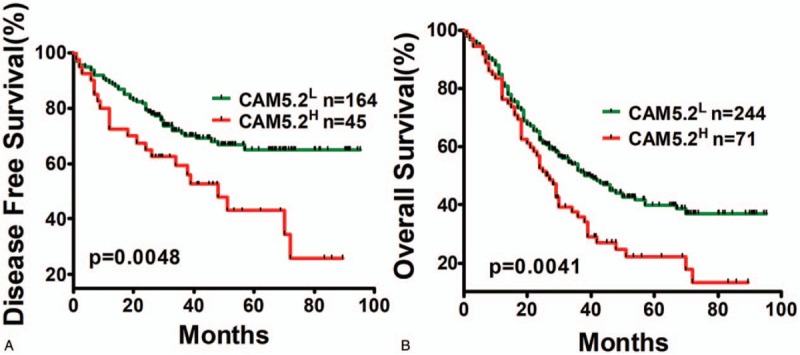
Relationship of ESCC CAM5.2 status to patients’ survival. Kaplan–Meier survival curves for (A) overall survival and (B) disease-free survival.

There was no association between clinicopathological parameters and CAM5.2 staining, whereas Kaplan-Meier analysis of 315 patients showed that strong CAM5.2 staining was associated with poor OS (*P* = .0041) (Fig. 4A) and poor DFS of ESCC patients (*P* = .0048) (Fig. 4B) after a 95.2-month follow-up. Also, in a multivariate Cox model, CAM5.2 expression was significantly associated with DFS and OS in ESCC patients (Table 2).

**Table 2:**
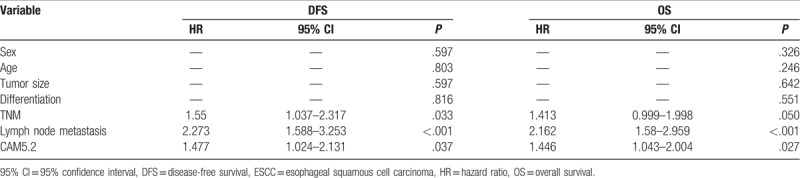
Multivariate Cox hazards analysis of DFS and OS in ESCC patients.

## 4 Discussion

The monoclonal antibody CAM5.2 was typically used to identify secretory epithelial (glandular epithelium) cells and epithelial tumors, such as colorectal cancer and pancreatic cancer. Additionally, CAM5.2 is generally accepted to be suitable for detection of metastatic breast cancer in (sentinel) lymph nodes.^[8]^ Rectal cancer patients with CAM5.2-positive cells in lateral lymph nodes should be regarded as having overt metastases.^[23]^ CAM5.2 is also a useful confirmatory stain in suspected metastatic adenocarcinoma to the brain, and the sensitivity and specificity of CAM5.2 in metastatic tumors was 100%.^[24]^ Furthermore, the multivariate Cox model showed significant association between CAM5.2 expression and DFS as well as OS in ESCC patients (Table 2), suggesting that CAM5.2 could be an independent prognostic factor for ESCC. Although CAM5.2 and ESCC have been characterized in certain studies, relatively little is known about the relationship between CAM5.2 staining status and ESCC prognosis. We reported for the first time that the strong staining of CAM5.2 is a poor prognostic marker for ESCC patients.

Tumor cells with strong CAM5.2 staining of ESCC still have typical pathological features of squamous epithelium. Immunostaining markers, such as CK5/6, can be used to distinguish squamous epithelium, whereas CAM5.2 and CK7 can be used for glandular epithelium.^[25]^ GEO data showed that CK5 and CK6 (CK6a, CK6b, and CK6c) mRNA levels decreased in ESCC, and CK8 mRNA levels increased, compared to their healthy counterparts, but CK7 mRNA levels showed no significant difference. Although the overall level of CK5 and CK6 mRNA levels decreased in ESCC samples, a study has shown that almost all ESCC highly expressed CK5/6 protein, only 1 of the 64 samples showed an immunohistochemical score of CK5/6 <10%.^[26]^ Thus, the expression difference of CK7 or CK8 was more meaningful.

Yamada et al^[27]^ noted that CK7 expression was a useful biomarker for predicting the outcome of stage I/IIA/IIB ESCC patients, and there were 28 positive staining cases (28/126, 22%). Oue et al^[28]^ pointed out CK7-positivity and receipt of adjuvant chemotherapy tended to be beneficial for ESCC patients with stage II/III disease, and 20 (9%) of 225 ESCC cases were positive for CK7. At present, few studies about the function of CK8 in ESCC were launched, and the relationship between the prognosis of CK8 and ESCC is not well understood. Our positive staining of CAM5.2 was 145 of 604 (24%), and CK7 positive staining was 59 of 594 (10%).^[29]^

There was no significant difference between the CAM5.2 staining status and the pathological features, probably because of the fact that it is a cytokeratin cocktail antibody. The significant difference may be observed between CAM5.2 staining status and clinicopathological features if CK7 or CK8 were detected respectively.

This study has some limitations: first, our data originated from a single-center database, and the patients in our study were predominantly Chinese, so the results may not be generalizable beyond a Chinese or Asian population. Second, this study was retrospective in nature, and blindness cannot be used in this study, which may inevitably induce investigator bias. Third, surgical specimens were used as a reference in this study, so only participants with operable ESCC were included. A future multicenter prospective study with a larger number of participants will be performed to verify these limitations.

In conclusion, high expression of cytokeratin CAM5.2 in ESCC is associated with poor prognosis.

## Author contributions

**Conceptualization:** ShuJin He, Jie Peng, Wei Wang.

**Data curation:** Jinguo Zhang, Renya Zhang, Wei Wang.

**Project administration:** Wei Wang.

**Software:** Lei Li, Ying Xu, Xiaoxiao Wu, Juan Yu.

**Visualization:** ShuJin He, Jie Peng, Jianli Liu.

**Writing – original draft:** ShuJin He, Jie Peng, Renya Zhang, Wei Wang.

**Writing – review & editing:** ShuJin He, Renya Zhang, Wei Wang.
